# Higher BMI is associated with vaginal microbiome alterations in women with PCOS

**DOI:** 10.1530/RAF-26-0051

**Published:** 2026-06-22

**Authors:** Marta Spochacz-Santoro, Anna Szeliga, Magdalena Durda-Masny, Katarzyna Morańska, Monika Englert-Golon, Wawrzyniec Sadowski, Maria Bocheńska, Swapnil Doijad, Bas E. Dutilh, Rose Brouns, Marlena Grabowska, Stefan Sajdak, Błażej Męczekalski, Anita Szwed

**Affiliations:** ^1^ Institute of Human Biology and Evolution, Faculty of Biology, Adam Mickiewicz University, Poznan, Poland; ^2^ Department of Gynecological Endocrinology, Poznan University of Medical Sciences, Poznan, Poland; ^3^ Department of Gynecology, Division of Gynecological Oncology, Gynecological Obstetric Clinical Hospital, Poznan University of Medical Sciences, Poznan, Poland; ^4^ Institute of Biodiversity, Ecology, and Evolution, Faculty of Biological Sciences, Cluster of Excellence Balance of the Microverse, Friedrich Schiller University Jena, Jena, Germany; ^5^ Theoretical Biology and Bioinformatics, Department of Biology, Science for Life, Utrecht University, Utrecht, The Netherlands; ^6^ Division of Gynecology, Gynecological Obstetric Clinical Hospital, Poznan University of Medical Sciences, Poznan, Poland

**Keywords:** polycystic ovary syndrome, PCOS, body mass index, BMI, gut microbiota, vaginal microbiota

## Abstract

**Abstract:**

Polycystic ovary syndrome (PCOS) is commonly associated with obesity and metabolic disturbances. Although gut and vaginal microbiome changes have been linked to PCOS, the independent role of body mass index in these alterations remains unclear. This study aimed to compare gut and vaginal microbiomes in women with PCOS and healthy controls, emphasizing the impact of body mass index. Seventy-five women were enrolled, including 55 with PCOS and 20 healthy controls. Participants were stratified into normal-weight (<25) and overweight (≥25) groups. Vaginal and anorectal swabs were analyzed using full-length 16S rRNA nanopore sequencing. Microbial diversity and composition were assessed with alpha diversity indices, principal component analysis, analysis of composition of microbes, PERMANOVA, and vaginal community state type classification. Without body mass index stratification, women with PCOS showed differences in several vaginal taxa compared with healthy controls. Within the PCOS cohort, overweight women exhibited higher vaginal alpha diversity, reduced *Lactobacillus* dominance, and enrichment of anaerobic taxa compared with normal-weight women with PCOS. Differences in vaginal microbial composition were also observed between healthy and PCOS women with normal body mass index. In contrast, gut microbiome alterations were limited and less consistent across analytical approaches. Vaginal community state type analysis revealed predominance of class IV communities in both healthy women and women with PCOS. These findings suggest that higher body mass index is associated with vaginal microbiome alterations in women with PCOS, although PCOS-related factors independent of body weight may also contribute to the observed microbial differences.

**Lay summary:**

Polycystic ovary syndrome is a common condition that affects hormones, fertility, and metabolism in women. Many women with this condition are also overweight, but it is not fully understood how body weight influences the bacteria living in the body. In this study, we compared bacteria present in vaginal and anorectal samples from women with and without polycystic ovary syndrome, while also considering body weight. We found that women with higher body weight had more noticeable changes in vaginal bacterial composition, including lower amounts of protective *Lactobacillus* bacteria and higher diversity of anaerobic bacteria. At the same time, some bacterial differences were also observed in women with polycystic ovary syndrome who had normal body weight. Changes in gut bacteria were smaller and less consistent. These findings suggest that both body weight and polycystic ovary syndrome may influence vaginal bacterial composition and should be considered in future studies of women’s reproductive health.

## Introduction

Polycystic ovary syndrome (PCOS) is an endocrine disorder globally affecting 9.8% of the population, according to the Rotterdam criteria (Rotterdam ESHRE/ASRM-Sponsored PCOS consensus workshop group 2004). To be diagnosed with PCOS, the patient should be affected by two out of three symptoms, such as hyperandrogenism, irregular or lack of ovulation, and polycystic ovary morphology (PCOM) (Amiri *et al.* 2025). Since the selected Rotterdam criteria can be differentiated and can appear in different configurations, there are four clinical subtypes of PCOS with different prevalence and metabolic characteristics (Wen *et al.* 2024). The etiology of PCOS is still uncertain. In general, the genetic predispositions are taken into account, especially epigenetic inheritance (Mimouni *et al.* 2021, Stener-Victorin *et al.* 2024), but also other components such as higher body mass index (BMI) (Deng *et al.* 2017).

Studies from the past decade have provided growing evidence of a potential relationship between gut microbiota dysbiosis and PCOS (Tremellen & Pearce 2012). The analysis of the gut microbiomes of women with PCOS compared with healthy controls showed significant differences in the diversity and phylogenetic composition (Lindheim *et al.* 2017, Ravat *et al.* 2024). Certain bacteria, such as *Escherichia* and *Shigella*, have been linked to dysbiosis in patients with PCOS, contributing to systemic inflammation and hormonal imbalances that aggravate clinical symptoms (Senthilkumar & Arumugam 2025). The research on the vaginal microbiome in PCOS remains limited. Existing studies indicate significant alterations in microbial composition in women with PCOS (Tu *et al.* 2020, Lu *et al.* 2021). The vaginal microbiome is considered highly dynamic. Changes in its composition and metabolic activity can occur rapidly in response to a variety of factors, such as lifestyle and sexual activity, gestational status, menstrual cycle, and contraceptive use (Lewis *et al.* 2017, Hong *et al.* 2020). The scarce results published so far suggest that there is a relationship between PCOS and vaginal dysbiosis, most likely caused by decreased vaginal *Lactobacillus* species and elevated pathogenic species, including *Streptococcus, Actinomyces, Prevotella, Gardnerella*, and *Mycoplasma* species, which occur alongside vaginal pH alterations (Pereira *et al.* 2024). However, when considering the composition of the microbiota in women with PCOS, the frequent coexistence of excess body weight should be taken into account. Most women with PCOS (up to 80%) are overweight or obese and frequently experience metabolic syndrome, insulin resistance, hyperinsulinemia, and diabetes mellitus. They may also develop dyslipidemia and are at increased risk of cardiovascular diseases. These metabolic disturbances may be associated with alterations in both gut and vaginal microbiota due to complex host–microbe interactions (Hussain *et al.* 2021). Microbial diversity has been shown to differ significantly between healthy and obese women (Allen *et al.* 2022), suggesting that BMI may be an important factor influencing microbiome composition in PCOS. However, detailed analyses of vaginal and gut microbiota in PCOS patients stratified by BMI remain scarce. Therefore, in this study, we aimed to examine vaginal and gut microbiome compositions in women with PCOS with normal body weight (BMI < 25) and excessive body mass (BMI > 25), compared with BMI-matched healthy controls.

## Materials and methods

### Ethical considerations

The study was conducted in accordance with the Declaration of Helsinki and approved by the Bioethics Committee of the Karol Marcinkowski Medical University in Poznań, Poland (Resolution No. 125/23). All participants provided written informed consent prior to sample collection.

### Study and control groups

A total of 55 premenopausal women with PCOS (18–49 years) and 20 healthy controls (20–51 years) were enrolled in the study. Patients with bacterial vaginosis and those using contraception and probiotics less than three months before the sample collection were excluded. Only patients with untreated PCOS and not receiving any other treatment were recruited. Participants were categorized by BMI as normal weight (BMI < 25 kg/m^2^; PCOS: *n* = 25, controls: *n* = 10) or overweight (BMI ≥ 25 kg/m^2^; PCOS: *n* = 30, controls:*n* = 10). PCOS was diagnosed according to the Rotterdam criteria (Rotterdam ESHRE/ASRM-Sponsored PCOS consensus workshop group 2004). All participants were recruited at the Gynecological and Obstetric Clinical Hospital, Poznan University of Medical Sciences, Poland. Cohort characteristics are presented in Table 1, with detailed metadata in Supplementary Table 1 (see section on Supplementary materials given at the end of the article). Adjustment for age, number of full-term pregnancies, and menstrual cycle day did not materially affect the observed associations.

**Table 1:** Basic characteristics of the study and control groups. Data are presented as *n* (%).

Characteristics	Study group (with PCOS)	Control group (healthy controls)
Age, years		
18–19	5 (9.09)	–
20–29	38 (69.09)	2 (10.00)
30–39	11 (20.00)	5 (25.00)
40–49	1 (1.82)	12 (60.00)
50–51	–	1 (5.00)
Body mass index		
<18.5	3 (5.45)	–
18.5–24.99	22 (40.00)	10 (50.00)
25.0–29.99	12 (21.82)	6 (30.00)
30.0–34.99	11 (20.00)	3 (15.00)
35.0–39.99	6 (10.91)	1 (5.00)
>40.0	1 (1.82)	–

### Data and sample collection

Data on weight, height, and parity were self-reported by participants. Swab samples were collected from the anorectal region and vagina by gynecologists using standardized sampling kits. Collected specimens were immediately preserved in a microbiome-dedicated buffer (eNAT® Nucleic Acid Collection and Preservation, Copan eNAT® System), consisting of 2 mL guanidine thiocyanate-based medium with nucleic acid-stabilizing properties. Samples were initially stored at −20°C and transported to the laboratory. For long-term preservation, samples were maintained at −80°C.

### DNA extraction

Total genomic DNA was extracted from 250 μL of swab samples in a stabilizing buffer using the DNeasy PowerSoil Pro Kit (Qiagen, Germany) according to the manufacturer’s instructions, with an additional step of 10 min incubation at 65°C before homogenization for better lysis. The homogenization step was performed using PowerBead Pro tubes and TissueLyser at a maximum speed for 2 min. Elution was performed in 50 μL solutions pre-heated to 60°C. DNA quantification was performed utilizing 1 μL of the samples and a Qubit 4 Fluorometer. Then, samples were stored at −20°C in the laboratory freezer.

### 16S rRNA amplicon sequencing

Full-length 16S rRNA amplicons (∼1.5 kb) were sequenced using the rapid barcoding 16S Barcoding Kit 1–24 (SQK-16S024, Oxford Nanopore Technologies, UK). Twenty-three patient samples and one negative control were pooled per run, each containing 10 ng of high-quality bacterial genomic DNA. Library preparation included 16S amplification with barcodes, magnetic bead cleanup (AMPure XP Beads), and ligation of sequencing adapters. Sequencing was performed on a MinION (with FLO-FLG001). The basic parameters for each run were as follows: i) time – 24 h, ii) voltage – 180 mV, iii) basecalling – fast, iv) minimum barcode score – 60, v) minimum read quality score – 7, and vi) output files – FAST5 format. Super basecalling and taxonomic assignments were subsequently conducted.

### Basecalling and amplicon sequence analysis

Super basecalling was performed using the Dorado basecaller (v0.6.0) with the dna_r9.4.1_e8_sup@v3.3 configuration. Reads were demultiplexed by primer sequence using a custom pipeline. Low-quality reads (Phred score < 10) and reads deviating ±100 bp from the expected 1,467 bp length were removed using Chopper v0.5.0. Exact matches to custom forward and reverse primers (and their reverse complements) were used to assign reads to their respective sample bins. Taxonomic classification at the species level was performed using Kraken2 v2.1.2 (database version: February 2023), and abundance tables were generated with KrakenTools v1.2. Rarefaction curves were constructed to assess potential biases arising from differences in sequencing depth, and sequencing saturation was defined as fewer than one newly detected taxon per 100 reads during consecutive subsampling. All samples reached a plateau, indicating that the sequencing depth was enough (Supplementary Fig. 1). All bioinformatics tools were used with default settings unless otherwise specified. The analysis script is available at https://github.com/SwapnilDoijad/veo_pipelines/blob/main/supplementary_scripts/0991_calculate_abundance_from_fastq.sbatch, version 258a814. The complete species-level abundance table, taxonomic assignments, and the abundance of individual taxa are available in a public repository (see the section titled Availability of data and materials).

### Quantification and statistical analysis

Samples with fewer than 200 reads were excluded from subsequent analyses. Data normalization methods varied depending on the specific analysis, as described below. All custom scripts are available at https://github.com/Greyeminences/microbiome_analysis.

Relative abundances were used to identify the top 20. For each sample, raw read counts were scaled to a sum of 1. The top *n* taxa were extracted per sample, rescaled to 1, and combined across samples to generate collective bar plots. Alpha diversity indices (Chao1, Shannon, and Simpson) were calculated using the phyloseq package in R. Statistical significance between groups was assessed using the Mann–Whitney U test. Principal component analysis (PCA) was performed in Python using the ‘sklearn.decomposition.PCA’ module, with points colored according to the variable of interest. Input data for PCA were raw counts transformed by the centered log-ratio. Group differences were evaluated via PERMANOVA, and multiple testing correction was applied using the Benjamini–Hochberg procedure to control the false discovery rate (FDR). Results with *P*-values <0.05 were considered significant and visualized using scatterplots in Python. Two strategies were used and compared for differential abundance analysis: analysis of composition of microbiomes with bias correction (ANCOM-BC) and a faster model based on the same method (fastANCOM). Parameters were as follows: prevalence cut 0.05; assay name ‘counts’; *P*_adj_method ‘holm’. A *P*-value less than 0.05 was considered significant.

## Results

### Vaginal microbiome composition and diversity in women with PCOS stratified by body mass index

**Figure 1:**
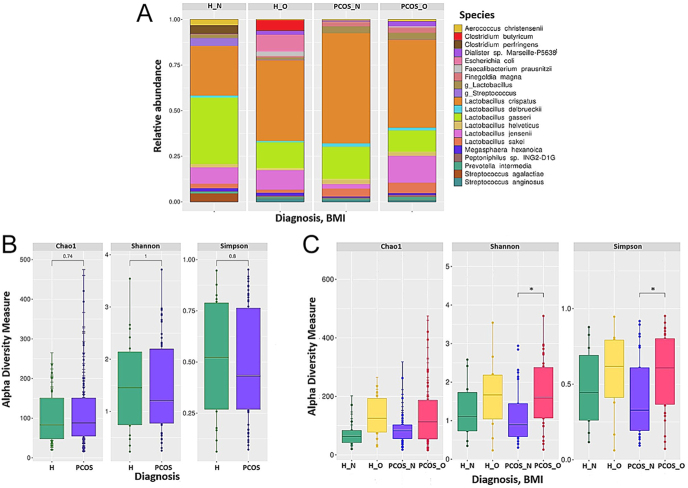
(A) Relative abundance of predominant vaginal taxa stratified by diagnosis and patient BMI. Abbreviations of patients’ designation: H_N – healthy, normal weight; H_O – healthy, overweight; PCOS_N – PCOS, normal weight; and PCOS_O – PCOS, overweight. (B) Comparison of alpha diversity metrics (Chao1, Shannon, and Simpson indices) between healthy individuals and patients with PCOS. Statistical significance was evaluated using the Mann–Whitney U test. (C) Alpha diversity comparisons between healthy and PCOS groups stratified by BMI, assessed using Chao1, Shannon, and Simpson indices. Statistical analyses were performed with the Mann–Whitney U test; significance was considered at *P* < 0.05.

In total, 1,024 bacterial taxa were detected across all patients in both niches. The vaginal microbiome was dominated by the genus *Lactobacillus*, with notable differences between PCOS patients and healthy controls. In the PCOS group, *Lactobacillus crispatus* accounted for 45% of the vaginal community, followed by *Lactobacillus gasseri* (12%) and *Lactobacillus jensenii* (7%), with additional contributions from *Lactobacillus sakei, Finegoldia magna*, and *Lactobacillus helveticus*. In the control group (healthy women), the abundance of individual taxa was distributed as follows: *L. crispatus* (29%), *L. gasseri* (21%), and *L. jensenii* (8%), along with *Escherichia coli, Clostridium butyricum, Clostridium perfringens*, and *Streptococcus agalactiae*. The relative abundance is presented in Fig. 1A.

The alpha diversity of vaginal microbiome (Fig. 1B) was similar across control and study groups. Stratification by BMI (Fig. 1C) revealed higher vaginal diversity in overweight versus normal-weight PCOS women (Shannon *P* = 0.012; Simpson *P* = 0.026), with a similar non-significant trend in healthy controls. Analysis of vaginal microbiome communities stratified by diagnosis and BMI revealed BMI-associated shifts in taxonomic composition. Among healthy overweight participants, the relative abundance of *E. coli* was increased by approximately seven percentage points, while *L. jensenii* and *L. crispatus* were enriched by 10 and 11 percentage points, respectively, compared with normal-weight women. In contrast, among women with PCOS, overweight individuals exhibited an approximately 9 percentage-point increase in *L. jensenii* and a 15 percentage-point reduction in *L. crispatus* relative to normal-weight PCOS patients. BMI was associated with distinct alterations in vaginal microbiome composition, with overweight linked to increased *L. jensenii* abundance in both groups and divergent patterns of *L. crispatus* between healthy and PCOS patients.

**Figure 2:**
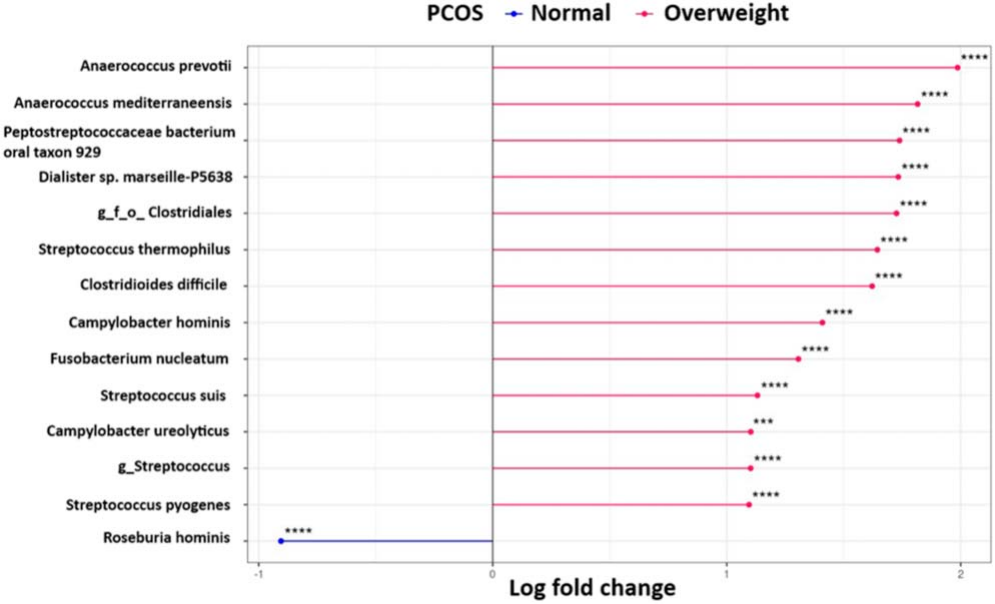
Results of ANCOM-BC of vaginal microbiomes in women diagnosed with PCOS with normal and excessive body mass; ‘g’, ‘f’, ‘o’ refer to the genus, family, and order, respectively. Positive log fold changes indicate taxa enriched in overweight individuals, and negative values indicate depleted taxa. Asterisks indicates the *q* value (FDR-adjusted *P* values) range: ***q < 0.001; ****q < 0.0001.

To further investigate the vaginal microbiome, differential abundance analyses were performed using ANCOM. All groups were compared with each other, taking into account the division of patients only according to diagnosis and diagnosis with BMI. Without BMI stratification, women with PCOS exhibited significantly higher relative abundance of several taxa, including *Tissierellia*, *Fusobacterium nucleatum*, *Lactobacillus salivarius*, *Lactobacillus amylophilus*, *Lactobacillus mucosae*, *Lactobacillus murinus*, *Lactobacillus hokkaidonensis*, and *Lactobacillus pentosus* (all q-values ranging from 2.1 × 10^−7^ to 0.0276) compared with healthy women. In contrast, healthy women showed enrichment of *Anaerococcus* spp. (*q* = 0.00027). Detailed data are available in Supplementary Fig. 2. In PCOS patients, 14 taxa were identified as significantly different between BMI categories (Fig. 2). Notably, in overweight PCOS patients, the majority of vaginal taxa exhibited positive log fold changes, indicating that elevated BMI was accompanied by enrichment of specific taxa. Compared with normal-weight PCOS women, the vaginal taxa with significantly increased abundance in overweight PCOS women included *A. prevotii, Anaerococcus mediterraneensis, Peptostreptococcaceae* bacterium oral taxon 929, *Dialister* sp., *Clostridiales, Streptococcus thermophilus, Clostridiales difficile, Campylobacter hominis, F. nucleatum, Streptococcus suis, Campylobacter ureolyticus*, *Streptococcus spp*., and *S. pyogenes*. In contrast, normal-weight women with PCOS showed higher relative abundance of *Roseburia hominis.* To assess whether the identified taxa represented sporadic findings, we additionally evaluated their prevalence across samples. Overall, these taxa were detected in 25 out of 75 samples (33%), including 8 samples from healthy women and 17 samples from women with PCOS, of whom 15 were overweight. ANCOM-BC comparing normal-weight healthy women and normal-weight women with PCOS identified differences in several vaginal taxa. Healthy controls showed higher relative abundance of multiple *Lactobacillus* species (*L. amylolyticus, L. amylophilus, L. gallinarum, L. kefiranofaciens, L. murinus, L. paracollinoides, and L. salivarius,* all *q* values < 0.05) as well as *Eubacterium eligens*. Detailed data are available in Supplementary Figs 4, 5, 6). In contrast, normal-weight women with PCOS were enriched in anaerobic taxa, including *Prevotella ruminicola*, *Paeniclostridium sordellii*, and *Dialister* sp., suggesting a shift toward a more anaerobic vaginal microbial profile. The largest number of differentially abundant vaginal taxa was observed between healthy normal-weight women and overweight women with PCOS. Compared with healthy controls, overweight women with PCOS showed enrichment of several anaerobic taxa, including *Fusobacterium nucleatum*, *Negativicoccus massiliensis*, and *Prevotella fusca*, together with depletion of selected taxa such as *Prevotella ruminicola* and *Veillonella rodentium*. However, vaginal community state type (CST) classification revealed generally similar community structures in both groups. Approximately 70% of healthy women and 60% of women with PCOS were classified as CST IV, characterized by a non-*Lactobacillus*-dominated and heterogeneous anaerobic microbiota (Supplementary Table 3).

**Figure 3:**
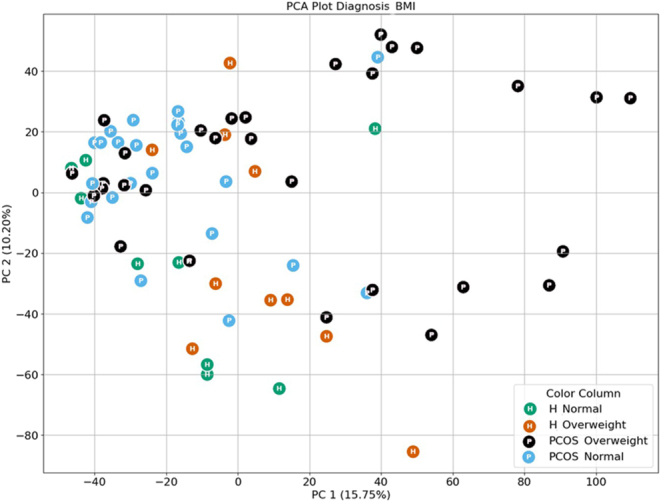
Principal component analysis (PCA) of vaginal microbiomes, comparing patients with PCOS and healthy controls across BMI categories.

To assess beta diversity, i.e. compositional differences between patients with PCOS and healthy controls across BMI categories, we used principal component analysis (PCA). For the vaginal dataset, PC1 and PC2 explained 15.75 and 10.20% of the total variance, respectively, accounting for 25.95% of the overall variability (Fig. 3). PCA revealed greater divergence in vaginal microbiome composition among overweight participants compared with their normal-weight counterparts, with the effect more pronounced in PCOS patients than in healthy controls, although a similar trend was observed in both groups. Within the PCOS cohort, overweight individuals exhibited the greatest within-group variability. PERMANOVA showed significant differences in community composition between several groups (Table 2). Significant differences were observed between healthy normal-weight women and all other groups, as well as between normal-weight and overweight participants overall (*q* ≤ 0.029). The difference was also detected between all healthy women and women with PCOS (FDR = 0.029), although the associated effect size was small (*R*^2^ = 0.0299). Within the PCOS group, vaginal microbiome composition differed between normal-weight and overweight women (FDR = 0.0225). In contrast, no significant difference was observed between overweight controls and overweight women with PCOS (FDR = 0.1910).

**Table 2:** PERMANOVA of vaginal beta diversity of microbiota. Statistically significant values are highlighted in bold.

Pair	F-value	*R^2^*	*P*-value	FDR
H_Normal vs H_Overweight	2.0344	0.1016	0.0140*	**0.0225**
H_Normal vs PCOS_Overweight	2.7689	0.0679	0.0190*	**0.0228**
H_Normal vs PCOS_Normal	2.7863	0.0779	0.0110*	**0.0225**
H_Overweight vs PCOS_Overweight	1.3547	0.0344	0.1910	0.1910
H_Overweight vs PCOS_Normal	3.3151	0.0913	**0.0010^‡^**	**0.0060**
PCOS_Overweight vs PCOS_Normal	2.9272	0.0523	0.0150*	**0.0225**
Normal vs overweight	3.4769	0.0455	**0.0030^†^**	**0.0030**
H vs PCOS	2.2493	0.0299	0.0290*	**0.0290**

* *P* < 0.05.

^†^ *P* < 0.01.

^‡^ *P* < 0.001.

### Association between PCOS, BMI, and gut microbiome composition and diversity

The gut microbiome exhibited comparable community structures between patients with PCOS and healthy controls in terms of the most abundant taxa, although it comprised a wider range of species with relatively lower proportions. The dominant species in both groups, representing approximately 35% of the community, included *Faecalibacterium prausnitzii*, *Peptostreptococcaceae* bacterium oral taxon 929, *F. magna, E. coli*, and *Peptoniphilus* sp. An additional 15% of the gut microbiome in both groups was represented by *Dialister marseille*, *C. hominis*, *Ezakiella massiliensis*, and *A. mediterraneensis*.

**Figure 4:**
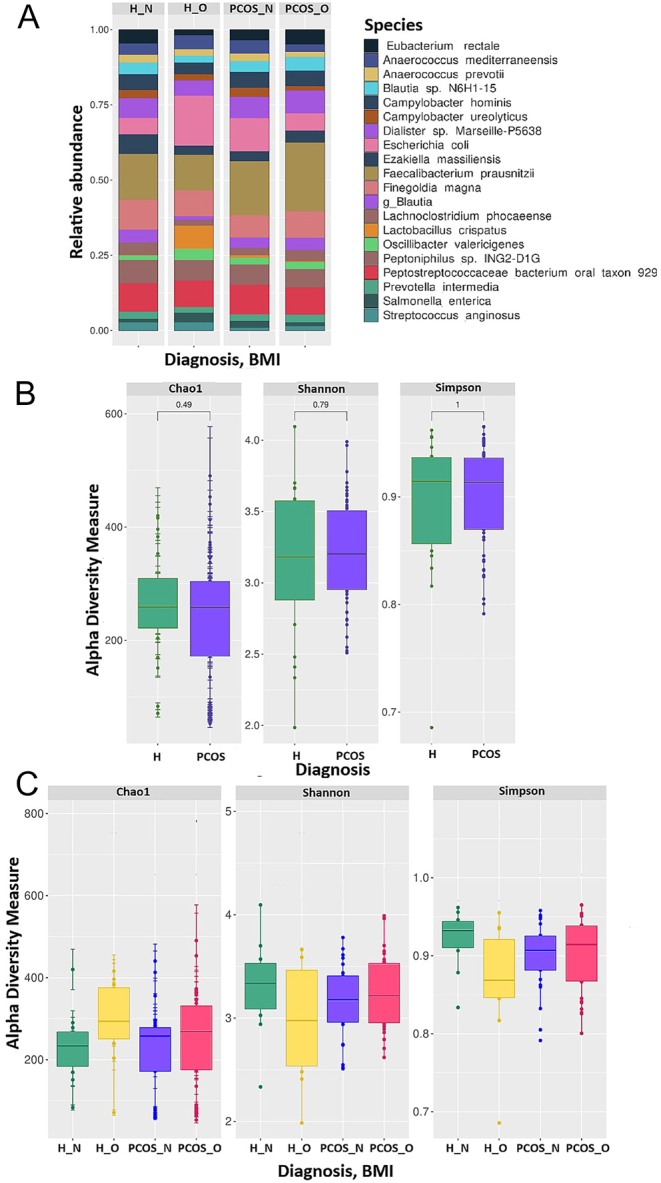
(A) Relative abundance of the most abundant gut taxa. Abbreviations of patients’ designation: H_N – healthy, normal weight; H_O – healthy, overweight; PCOS_N – PCOS, normal weight; and PCOS_O – PCOS, overweight. (B) Alpha diversity comparisons across study groups. Diversity indices (Chao1, Shannon, and Simpson) in women with PCOS versus healthy controls. For statistical analysis, the Mann–Whitney U test was used. (C) Alpha diversity indices stratified by BMI categories, comparing normal-weight and overweight participants within PCOS and control groups. The boxplots show median and interquartile range. For statistical analysis, the Mann–Whitney U test was used.

The relative abundances of the top taxa in the gut microbiome stratified by BMI are shown in Fig. 4A. Gut microbiomes were similar across groups in their most abundant taxa.

Gut microbiome diversity showed no significant differences, except for a tendency toward higher gut diversity in normal-BMI controls (Simpson *P* = 0.054). Results are summarized in Fig. 4C.

**Figure 5:**
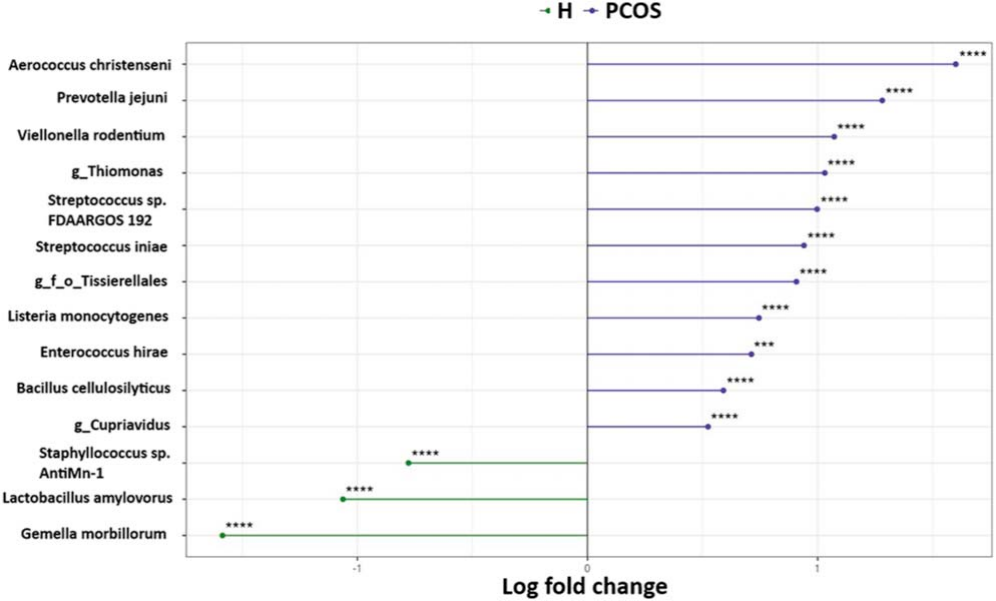
Results of ANCOM-BC of gut microbiomes in healthy women and women diagnosed with PCOS; ‘g’, ‘f’, ‘o’ refer to the genus, family, and order, respectively. Positive log fold changes indicate taxa enriched in PCOS individuals, and negative values indicate depleted taxa (***q < 0.001, ****q < 0.0001).

ANCOM-BC of gut microbiomes (Fig. 5) showed statistically significant differences in samples obtained from patients diagnosed with PCOS compared with healthy controls. Taxa specified for PCOS were *Aerococcus christensenii, Prevotella jejuni, Veillonella rodentium, Thiomonas* sp.*, Streptococcus* sp. *FDAARGOS 192, Streptococcus iniae, Tissierellales*, *Listeria monocytogenes, Enterococcus hirae, Bacillus cellulosilyticus,* and *Cupriavidus* sp. In contrast, the gut microbiomes of healthy patients contained *Staphylococcus* sp. *AntiMn-1, Lactobacillus amylovorus,* and *Gemella morbillorum.* When BMI and diagnosis were considered together, several taxa-level differences were identified in the gut microbiome. However, no strong or consistent global gut microbiome shifts were observed. Compared with healthy normal-weight women, normal-weight women with PCOS showed higher relative abundance of *Prevotella jejuni* (*q* = 0.004), Enterobacterales-related taxa (*q* = 0.0058), and *L. jensenii* (*q* = 0.044). In contrast, healthy normal-weight women were enriched in *Sphingomonas*, *Blautia sp.*, *Parabacteroides distasonis*, and *Prevotella denticola* (all *q* ≤ 0.035). Comparison between healthy normal-weight women and overweight women with PCOS revealed increased abundance of *Lactobacillus jensenii* and *Veillonella rodentium*, together with reduced abundance of *Bacteroides coprosuis* and *Sphingomonas* (all *q* ≤ 0.039). In contrast to the vaginal microbiome, only two taxa differed between normal-weight and overweight women within the PCOS cohort (*Enterobacter* sp. and *Enterobacter cloacae*), suggesting that BMI-related gut microbiome alterations in PCOS may be relatively limited. Detailed results are presented in Supplementary Figs 7, 8, 9.

For the gut PCA dataset, PC1 and PC2 accounted for 8.13 and 7.20% of the total variance, respectively (15.33% combined). Samples from all groups clustered closely, indicating similar gut microbiome composition regardless of diagnosis or BMI category. In PERMANOVA, no significant differences in gut microbiome composition were observed between groups after FDR correction. Although a nominal difference was detected between overweight controls and overweight women with PCOS (*P* = 0.025), this did not remain significant after adjustment for multiple testing (FDR = 0.15) and was associated with a small effect size. The PCA plot, together with PERMANOVA for gut data, is attached in the Supplementary data, Fig. 3 and Table 2, respectively.

## Discussion

Our study aimed to describe the vaginal and gut microbiome profiles of patients with PCOS, with and without excessive body mass. Our results suggested that both BMI and PCOS status may be associated with vaginal microbiome composition, although the observed differences were generally modest. In contrast, no strong or consistent global shifts were observed in the gut microbiome. The role of gut microbiota diversity in PCOS remains uncertain, and available results are contradictory. Studies by Eyupoglu *et al.* (2020), Li *et al.* (2020), Lüll *et al.* (2021), and Liang *et al.* (2021), among others, demonstrated no differences in diversity indices between the studied groups of women with and without PCOS. However, some researchers have shown decreased alpha diversity in PCOS patients (Jobira *et al.* 2020, Liang *et al.* 2020, Suturina *et al.* 2022) or differences in beta diversity analyses (Lindheim *et al.* 2017, Jobira *et al.* 2020). Most studies associated changes in the microbiome with PCOS, whereas heterogeneity between studies in terms of ethnicity, study protocol, sample size, or BMI makes it difficult to draw consistent conclusions (Sola-Leyva *et al.* 2023). Despite differences in diversity indices, some genera appear to be less prevalent in the gut microbiota of women with PCOS, including *Blautia* (Li *et al.* 2020, Dong *et al.* 2021), *Lactobacillus* spp. (Liu *et al.* 2017), *Faecalibacterium* (Zhang *et al.* 2019, Chu *et al.* 2020), and *Bifidobacterium* spp. (Zhang *et al.* 2019). Taxa reported to be more prevalent in women with PCOS include *Bacteroides* spp. (Zeng *et al.* 2019, Chu *et al.* 2020, Haudum *et al.* 2020), *Escherichia/Shigella* (Chu *et al.* 2020), and *Parabacteroides* spp. (Zhang *et al.* 2019, Chu *et al.* 2020).

In our study, ANCOM identified several gut taxa that differed between selected groups. However, these differences were not accompanied by significant global community shifts after FDR correction in PERMANOVA, and the observed effect sizes were small. Only two taxa differed between normal-weight and overweight women within the PCOS cohort, suggesting that BMI-associated gut microbiome alterations in PCOS may be relatively limited. Therefore, the observed gut microbiome differences should be interpreted cautiously.

In contrast, more pronounced differences were observed in the vaginal microbiome. Within the PCOS cohort, overweight women showed enrichment of multiple anaerobic taxa compared with normal-weight women with PCOS. Several taxa enriched in women with PCOS, including *Fusobacterium nucleatum* and *Tissierellia*, have previously been associated with anaerobic vaginal environments and microbial dysbiosis. At the same time, healthy women were characterized by higher relative abundance of several *Lactobacillus* species. Interestingly, differences in vaginal microbial composition were also observed between healthy and PCOS women with normal BMI. Healthy women were characterized by higher abundance of several *Lactobacillus* species, whereas women with PCOS showed enrichment of anaerobic taxa. This suggests that PCOS-associated vaginal microbiome alterations may not be explained exclusively by excess body weight. Overweight and obesity in women with PCOS have previously been linked to alterations in the vaginal microbiome, including decreased *Lactobacillus* dominance and increased abundance of anaerobic bacteria (Ceccarani *et al.* 2019, Chen *et al.* 2021, Garg *et al.* 2023). Some taxa identified in our study, including members of the *Prevotella* genus, have previously been associated with obesity and altered microbial communities (Hu *et al.* 2015, Larsen 2017). On the other hand, a UK-based case–control study found that obese women were less likely to have a *Lactobacillus*-dominant vaginal microbiota, with decreased abundance of *L. crispatus* and higher relative abundance of *Dialister* species, *Anaerococcus* vaginalis, and *Prevotella timonensis* than healthy-weight women (Raglan *et al.* 2021), which is partially consistent with our findings. However, CST analysis revealed predominance of CST IV communities in both healthy women and women with PCOS, suggesting that the observed vaginal microbiome alterations are relatively subtle and heterogeneous rather than representing completely distinct community structures. Understanding these associations may have implications for future diagnostic and therapeutic approaches in PCOS and obesity. Previous studies suggest that interactions between the microbiome and PCOS may be bidirectional, and modulation of the vaginal microbiome has been proposed as a potential supportive strategy for improving reproductive and metabolic health (Zheng *et al.* 2024).

Taken together, our findings suggest that higher BMI may contribute substantially to vaginal microbiome alterations in women with PCOS, particularly toward a more anaerobic microbial profile. At the same time, differences observed between healthy and PCOS women with normal BMI indicate that PCOS-related factors independent of body weight may also influence the vaginal microbiome. The lower reproductive tract microbiome may therefore be more susceptible to metabolic and hormonal changes associated with PCOS and excess body mass than the gut microbiome, although this interpretation remains tentative due to the limitations listed below.

### Limitations

Several limitations should be considered when interpreting the results of this study. First, the relatively small sample size, particularly after stratification by BMI, limits the statistical power to detect subtle differences in microbiome composition, especially in the anorectal samples. Larger cohorts are needed to confirm the observed trends and improve generalizability. The age imbalance between control group and PCOS group could influence the vaginal microbiome composition. Although age was included as a covariate in the analyses, residual confounding cannot be excluded. Second, the cross-sectional design prevents causal inference. Although associations between BMI, PCOS, and microbiome composition were identified, it cannot be determined whether excess body weight drives microbial changes, whether microbiome alterations contribute to metabolic or hormonal dysregulation, or whether both are shaped by shared underlying mechanisms. Longitudinal studies are necessary to clarify temporal and causal relationships. Third, several factors known to influence microbiome composition were not fully controlled, including diet, alcohol consumption, smoking, physical activity, or sexual behavior. These potential confounders may have contributed to inter-individual variability and influenced microbial profiles. Fourth, several methodological limitations should be considered. The relatively low read-depth threshold (200 reads) may have limited the stability of diversity estimates, particularly for low-abundance taxa. In addition, some taxa identified at the species level are uncommon in the analyzed niches and should therefore be interpreted cautiously. Although full-length 16S rRNA sequencing improves taxonomic resolution, misclassification of low-abundance taxa or limitations of reference databases cannot be fully excluded. Moreover, PERMANOVA results should be interpreted with caution because dispersion testing was not included in the original analysis, and differences in within-group variability may have influenced some of the observed associations. Fifth, BMI was used as the primary measure of adiposity and metabolic status, although it does not capture body fat distribution, insulin resistance, or other metabolic parameters that may be more directly linked to microbiome variation. Future studies incorporating detailed metabolic and hormonal biomarkers would allow more precise interpretation of host–microbiome interactions. Finally, participants were recruited from a single clinical center in Poland, which may limit the external validity of the findings across different populations, ethnic groups, and healthcare settings. Multicenter and population-based studies will be important to validate and extend these results.

Despite these limitations, this study provides meaningful evidence that BMI is associated with vaginal microbiome alterations in women with PCOS and underscores the importance of accounting for body weight in future microbiome research in this field.

## Conclusion

Our findings suggest that vaginal microbiome composition in women with PCOS is associated with both BMI and PCOS diagnosis, although the observed differences were generally modest and heterogeneous. The most pronounced vaginal microbiome alterations were observed in overweight women diagnosed with PCOS, who showed enrichment of anaerobic taxa and reduced *Lactobacillus* dominance compared with healthy women. At the same time, differences detected between healthy and PCOS women with normal BMI indicate that factors related to PCOS itself may also contribute to vaginal microbial variation independently of body weight.

In contrast, gut microbiome differences were limited and less consistent across analytical approaches, suggesting that the lower reproductive tract microbiome may be more sensitive to metabolic and hormonal disturbances associated with PCOS and increased body mass than the intestinal niche. Due to the cross-sectional design and methodological limitations of the study, these findings should be interpreted cautiously and require confirmation in larger, longitudinal cohorts.

## Supplementary materials



## Declaration of interest

The authors declare that there is no conflict of interest that could be perceived as prejudicing the impartiality of the work reported.

## Funding

This research did not receive any specific grant from any funding agency in the public, commercial, or not-for-profit sector.

## Author contribution statement

MS and AS contributed to conceptualization and methodology. AS, MG, MD, KM, MB, and WS contributed to investigation. MD, MS, ASzw, and ME contributed to data curation. KM, SD, RB, and BD contributed to formal analysis. AS, ME, SS, BM, and ASzw provided resources. MS contributed to writing of the original draft. ASzw, ME, BM, and SS contributed to supervision. All authors contributed to manuscript review and editing.

## Availability of data and materials

Additional results generated in this study are deposited in the Supplementary data. Any additional data are accessible from the lead contact upon request.

- Sequencing data that support the findings of this study have been deposited in NCBI SRA and can be accessed with BioProject identifier PRJNA1373157. Summary of sequencing depth within each niche after filtering is presented in Supplementary Table 4.
- The script used for amplicon sequencing analysis is accessible at https://github.com/SwapnilDoijad/veo_pipelines/blob/main/supplementary_scripts/0991_calculate_abundance_from_fastq.sbatch, version 258a814.
- The repository of created scripts for data analysis is accessible at https://github.com/Greyeminences/microbiome_analysis.
- Processed species-level abundance tables and the abundance of individual taxa are available for reviewers at https://zenodo.org/records/18835531?preview=1&token=eyJhbGciOiJIUzUxMiJ9.eyJpZCI6mVmNGQ4YzU3LTMzMGQtNDE4Yy1hYjQyLWY0NTE4NzYxN2E3MyIsImRhdGEiOnt9LCJyYW5kb20iOiJmOTY1NGNlMDg2M2IxYTAzN2JkNzU3YjgxYzBmYTllMCJ9.WA93pY2wxhWy86rkMAZeW74L_V5NlANInSxKgZHkegD7ySoK3ZhJaALbutsiaokDkQWc89jdqntwxeRFnWHCDw.
- Any additional information required to reanalyze the data reported in this paper is available from the lead contact upon request.
