# Supplementary material for: Higher BMI is associated with vaginal microbiome alterations in women with PCOS
Source: Reprod Fertil. 2026 Jun 22;7(2):RAF260051. doi: 10.1530/RAF-26-0051 (PMC13292985; doi:10.1530/RAF-26-0051)
Supplement: Supplementary file 1 [file supplementary_materials.pdf]

Supplementary Table 1. Detailed characteristic of the study and control groups

Patient code	Source name	Full term pregnancies	Age	Menopause	Body weight, kg	Body height, cm	BMI	BMI category	Diagnosis
patient_288	vagina_986	0	23	before	42	155	17,4818	Normal	PCOS
patient_344	vagina_978	0	26	before	57,8	177	18,4494	Normal	PCOS
patient_280	vagina_993	0	20	before	51	166	18,5078	Normal	PCOS
patient_326	vagina_890	0	27	before	51	163	19,1953	Normal	PCOS
patient_379	vagina_921	1	32	before	61	178	19,2526	Normal	PCOS
patient_306	vagina_922	0	19	before	59	172	19,9432	Normal	PCOS
patient_310	vagina_851	0	21	before	55,6	165	20,4224	Normal	PCOS
patient_295	vagina_845	0	26	before	53	161	20,4467	Normal	PCOS
patient_378	vagina_920	0	21	before	59	167	21,1553	Normal	PCOS
patient_336	vagina_988	0	21	before	65	175	21,2245	Normal	PCOS
patient_308	vagina_865	1	34	before	62	170	21,4533	Normal	PCOS
patient_317	vagina_855	1	24	before	56	160	21,875	Normal	PCOS
patient_337	vagina_898	0	22	before	69	177	22,0243	Normal	PCOS
patient_286	vagina_835	0	29	before	63,5	168	22,4986	Normal	PCOS
patient_345	vagina_927	0	24	before	60	162	22,8624	Normal	PCOS
patient_327	vagina_846	1	31	before	66	169	23,1084	Normal	PCOS
patient_339	vagina_878	0	29	before	61	162	23,2434	Normal	PCOS
patient_349	vagina_991	0	30	before	72,2	176	23,3084	Normal	PCOS
patient_281	vagina_826	0	20	before	70,6	173	23,5892	Normal	PCOS
patient_296	vagina_847	0	24	before	64,5	165	23,6915	Normal	PCOS
patient_319	vagina_906	0	37	before	58,8	157	23,8549	Normal	PCOS
patient_324	vagina_887		27	before	64,3	164	23,9069	Normal	PCOS

patient_309	vagina_821	0	26	before	65,8	164	24,4646	Normal	PCOS
patient_282	vagina_992	0	18	before	63	160	24,6094	Normal	PCOS
patient_322	vagina_870	0	24	before	68	165	24,977	Normal	PCOS
patient_313	vagina_874	0	28	before	67,4	163	25,3679	Overweight	PCOS
patient_351	vagina_842	0	38	before	69,3	165	25,4546	Overweight	PCOS
patient_376	vagina_995	0	26	before	77	171	26,3329	Overweight	PCOS
patient_312	vagina_914	0	26	before	76	168	26,9274	Overweight	PCOS
patient_307	vagina_924	0	19	before	78,5	170	27,1626	Overweight	PCOS
patient_320	vagina_883	1	31	before	80,5	172	27,2107	Overweight	PCOS
patient_318	vagina_881	0	30	before	82,5	172	27,8867	Overweight	PCOS
patient_298	vagina_915	2	36	before	78	167	27,968	Overweight	PCOS
patient_334	vagina_862	0	22	before	75	163	28,2284	Overweight	PCOS
patient_377	vagina_918	0	21	before	78	165	28,6501	Overweight	PCOS
patient_328	vagina_892	0	26	before	85	170	29,4118	Overweight	PCOS
patient_343	vagina_904	0	25	before	83,7	168	29,6556	Overweight	PCOS
patient_283	vagina_828	0	34	before	87	170	30,1038	Overweight	PCOS
patient_302	vagina_852	0	19	before	78	160	30,4688	Overweight	PCOS
patient_305	vagina_823	0	19	before	92	173	30,7394	Overweight	PCOS
patient_331	vagina_987	0	26	before	103	181	31,4398	Overweight	PCOS
patient_315	vagina_919	0	20	before	92	170	31,8339	Overweight	PCOS
patient_381	vagina_926	0	32	before	94	170	32,526	Overweight	PCOS
patient_380	vagina_925	0	20	before	88	164	32,7186	Overweight	PCOS
patient_316	vagina_923	0	27	before	92	167	32,9879	Overweight	PCOS
patient_333	vagina_889	0	22	before	85	160	33,2031	Overweight	PCOS
patient_375	vagina_990	0	20	before	107	179	33,3947	Overweight	PCOS
patient_347	vagina_837	0	28	before	93	166	33,7495	Overweight	PCOS

patient_323	vagina_886	1	41	before	66,9	137	35,6439	Overweight	PCOS
patient_291	vagina_841	0	25	before	92	160	35,9375	Overweight	PCOS
patient_321	vagina_820	0	22	before	120	182	36,2275	Overweight	PCOS
patient_329	vagina_894	0	20	before	99	164	36,8085	Overweight	PCOS
patient_338	vagina_900	0	23	before	83,5	150	37,1111	Overweight	PCOS
patient_300	vagina_853	0	23	before	104,3	165	38,3104	Overweight	PCOS
patient_285	vagina_833	0	25	before	113	167	40,5178	Overweight	PCOS
patient_031	vagina_63	2	37	before	62	170	21,4533	Normal	H
patient_091	vagina_208	2	40	before	62	164	23,0518	Normal	H
patient_080	vagina_241	0	26	before	57	159	22,5466	Normal	H
patient_088	vagina_362	3	44	before	65	163	24,4646	Normal	H
patient_193	vagina_606	1	34	before	84	165	30,8540	Overweight	H
patient_181	vagina_608	2	33	before	73	169	25,5593	Overweight	H
patient_194	vagina_610	0	28	before	75	161	28,9341	Overweight	H
patient_216	vagina_618	0	48	before	75	170	25,9516	Overweight	H
patient_217	vagina_622	2	44	before	102	168	36,1395	Overweight	H
patient_210	vagina_645	2	44	before	75	164	27,8852	Overweight	H
patient_093	vagina_651	0	43	before	46	153	19,6506	Normal	H
patient_184	vagina_652	3	48	before	65	165	23,8751	Normal	H
patient_220	vagina_681	2	48	before	70	170	24,2215	Normal	H
patient_213	vagina_694	2	51	before	56,5	159	22,3488	Normal	H
patient_237	vagina_704	2	44	before	80	160	31,2500	Overweight	H
patient_247	vagina_752	2	39	before	67	167	24,0238	Normal	H
patient_269	vagina_794	2	42	before	73	168	25,8645	Overweight	H
patient_382	vagina_963	0	45	before	96	170	33,2180	Overweight	H
patient_384	vagina_967	2	47	before	78	163	29,3575	Overweight	H

patient_249	vagina_971	1	36	before	66	163	24,8410	Normal	H

Supplementary Table 2. PERMANOVA analysis of gut beta diversity of microbiota. Statistically significant values are highlited in bold; *p* <0.05*

**GUT**				
Pair	F-value	R-squared	P-value	FDR
H_Overweight vs PCOS_Overweight	1,9487	0,0488	**0,0250**	0,1500
H_Overweight vs PCOS_Normal	1,2368	0,0361	0,2360	0,7080
H_Normal vs PCOS_Overweight	0,9070	0,0233	0,5590	0,8000
H_Normal vs H_Overweight	0,7977	0,0424	0,6770	0,8000
PCOS_Normal vs PCOS_Overweight	0,7577	0,0141	0,7960	0,8000
H_Normal vs PCOS_Normal	0,7714	0,0228	0,8000	0,8000
Normal vs Overweight	0,5470	0,0074	0,9910	0,9910
H vs PCOS	1,5630	0,0210	0,0680	0,0680

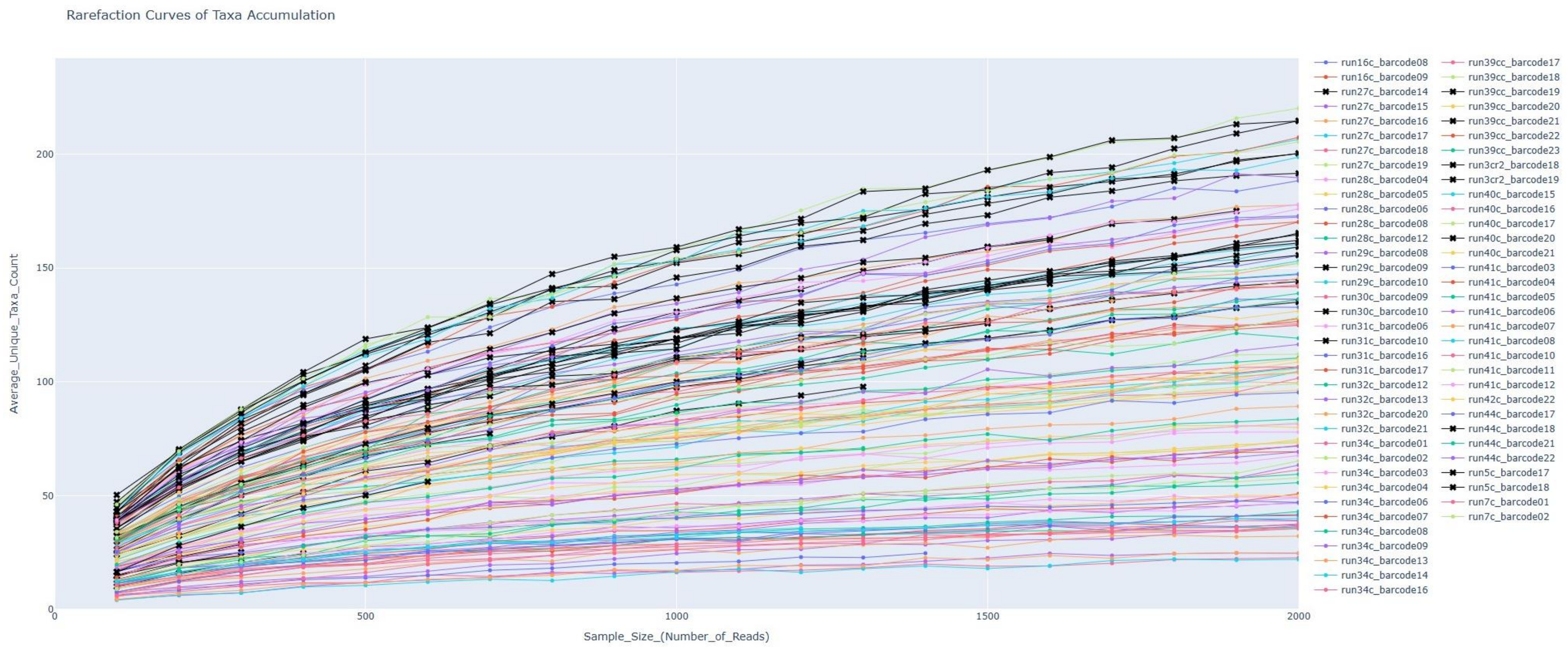

Supplementary Figure 1. Rarefaction curves from each sequencing run setup.

Colored lines with dot symbols indicate samples that got saturated, unsaturated samples are black with 'x' symbol.

Rarefaction curves were constructed to assess potential biases from differences in sequencing depth, and the OTU plateau was defined as fewer than one novel OTU per 100 reads during consecutive subsampling.

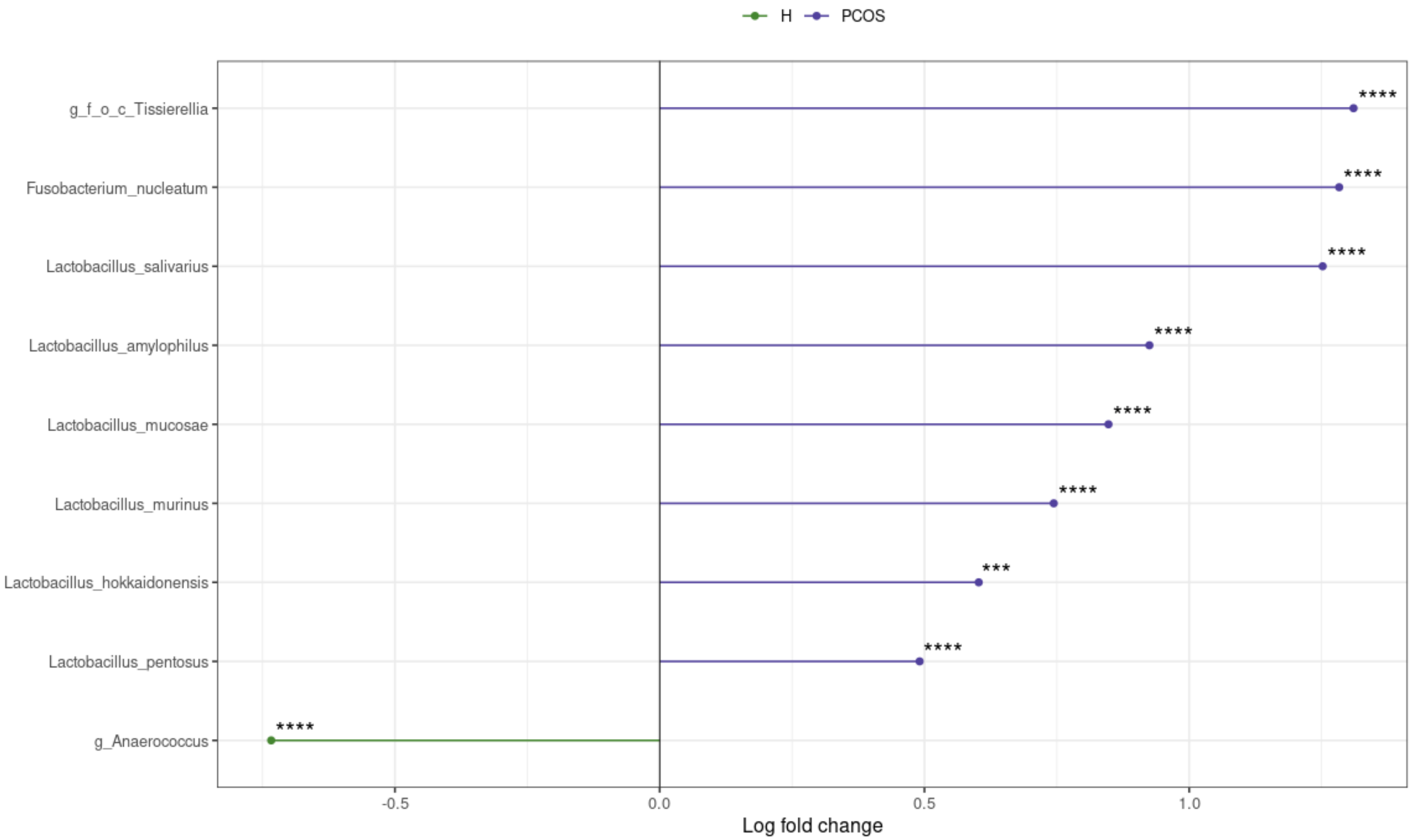

Supplementary Figure 2. Results of ANCOM-BC analysis of vaginal microbiomes in healthy women compared with women diagnosed with PCOS; g', 'f', 'o' refer to the genus, family and order, respectively. Positive log fold changes indicate taxa enriched in overweight individuals, negative values indicate depleted taxa. Asterisks indicates the q value (FDR-adjusted p values) rage;*q<0.001; ****q<0.0001**

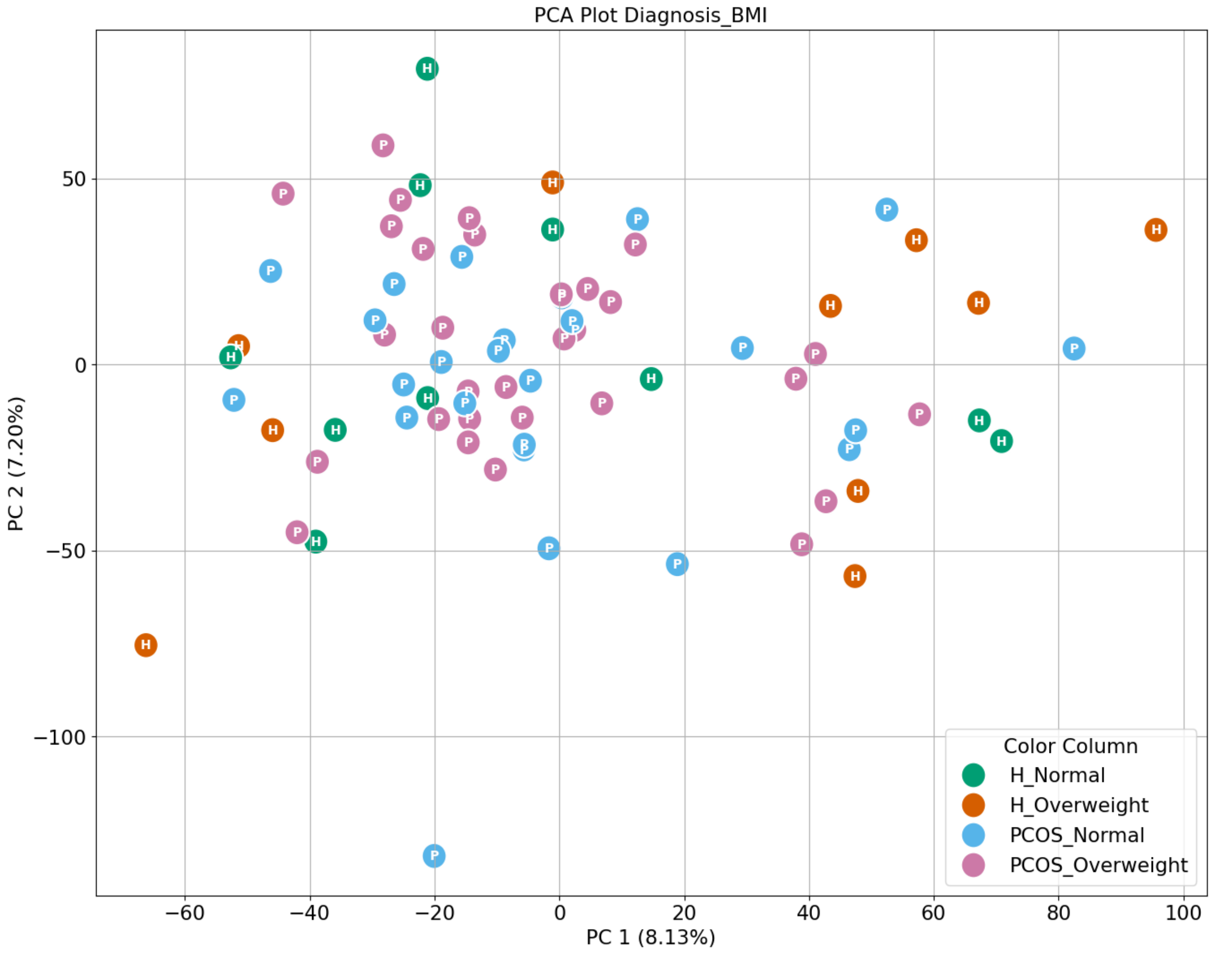

Supplementary Figure 3. Principal component analysis (PCA) of gut microbiomes, comparing patients with PCOS and healthy controls across BMI categories.

Supplementary Table 3. Results of vaginal microbiome classification to vaginal community types (CST)

	Samples of vaginal microbiome type (CST)					**Total numer of samples**	Percentage of samples in vaginal microbiome type (CST,%)				
**Diagnosis**	I	II	III	IV	V		I	II	III	IV	V
H_Normal	4	0	0	6	0	**10**	40	0	0	60	0
H_Overweight	2	0	0	8	0	**10**	20	0	0	80	0
PCOS_Normal	11	0	0	14	0	**25**	44	0	0	56	0
PCOS_Overweight	11	0	0	19	0	**30**	36,7	0	0	63,3	0
H	6	0	0	14	0	**20**	30	0	0	70	0
PCOS	22	0	0	33	0	**55**	40	0	0	60	0
Summary	56	0	0	94	0						

Supplementary Table 4. Summary of sequencing depth within each niche after filtering

VAGINA				
**Diagnosis**	**n_samples**	**range**	**mean_reads**	**median_reads**
H	20	217.0 - 18856.0	4716,98	2334
PCOS	55	216.0 - 19186.0	4243,08	3113
**Diagnosis_BMI**	**n_samples**	**range**	**mean_reads**	**median_reads**
H_Normal	10	217.0 - 9271.0	2256,7	1116,5
H_Overweight	10	225.0 - 18856.0	7177,25	5730
PCOS_Normal	25	253.0 - 13669.0	4447,96	3515
PCOS_Overweight	30	216.0 - 19186.0	4072,35	3010
**GUT**				
**Diagnosis**	**n_samples**	**range**	**mean_reads**	**median_reads**
H	20	360.0 - 17055.0	7356,55	6932
PCOS	55	385.5 - 8889.0	3073,18	2749
**Diagnosis_BMI**	**n_samples**	**range**	**mean_reads**	**median_reads**
H_Normal	10	360.0 - 14691.0	6740	5028,5
H_Overweight	10	957.0 - 17055.0	7973,1	9186
PCOS_Normal	25	436.0 - 8889.0	3205,28	3091
PCOS_Overweight	30	385.5 - 7113.0	2963,1	2593

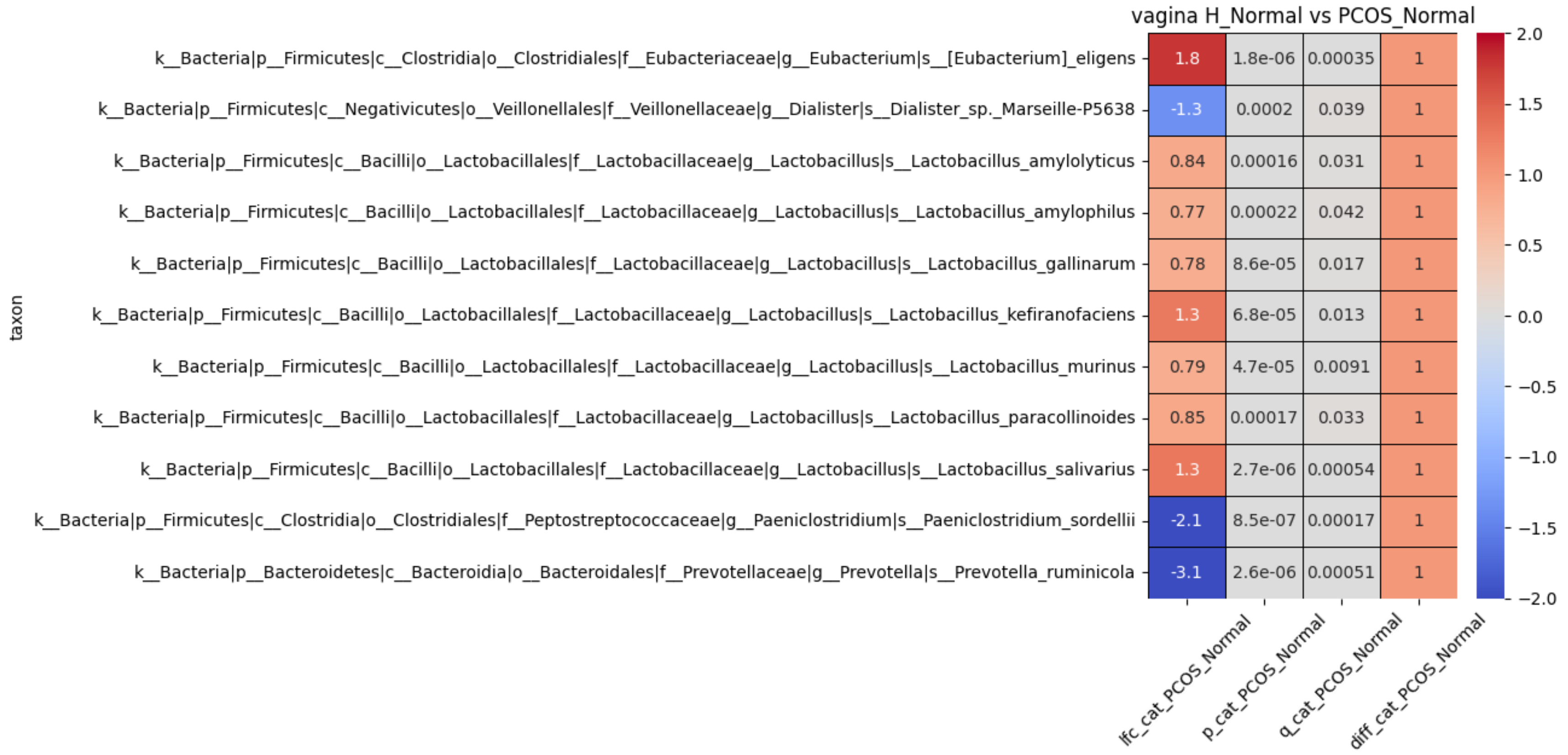

.

Supplementary Figure 4. ANCOM-BC differential abundance analysis of vaginal taxa between healthy normal-weight women and normalweight women with PCOS. Only significantly different taxa (FDR-adjusted q<0.05) are shown

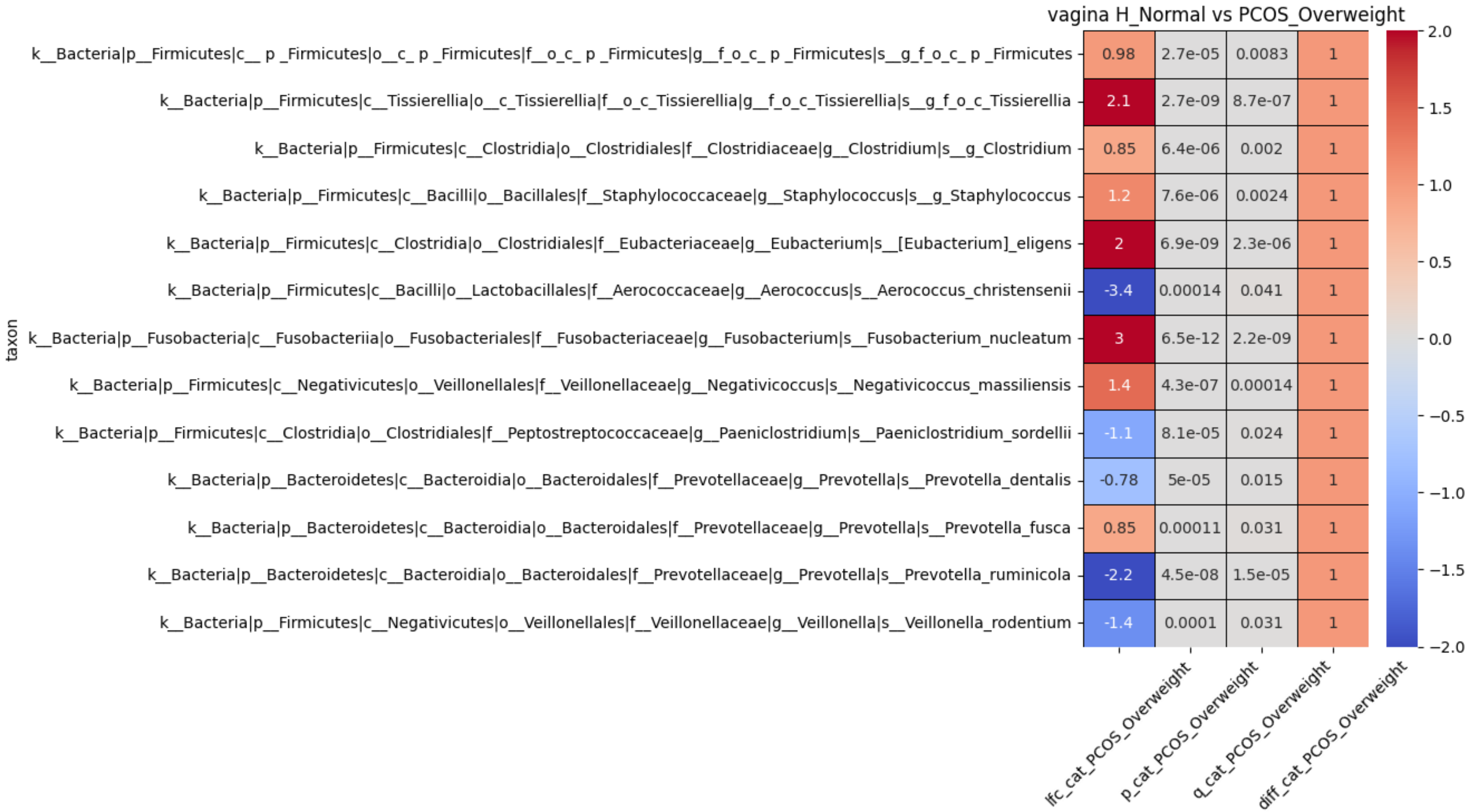

Supplementary Figure 5. ANCOM-BC differential abundance analysis of vaginal taxa between healthy normal-weight women and overweight women with PCOS. Only significantly different taxa (FDR-adjusted q<0.05) are shown

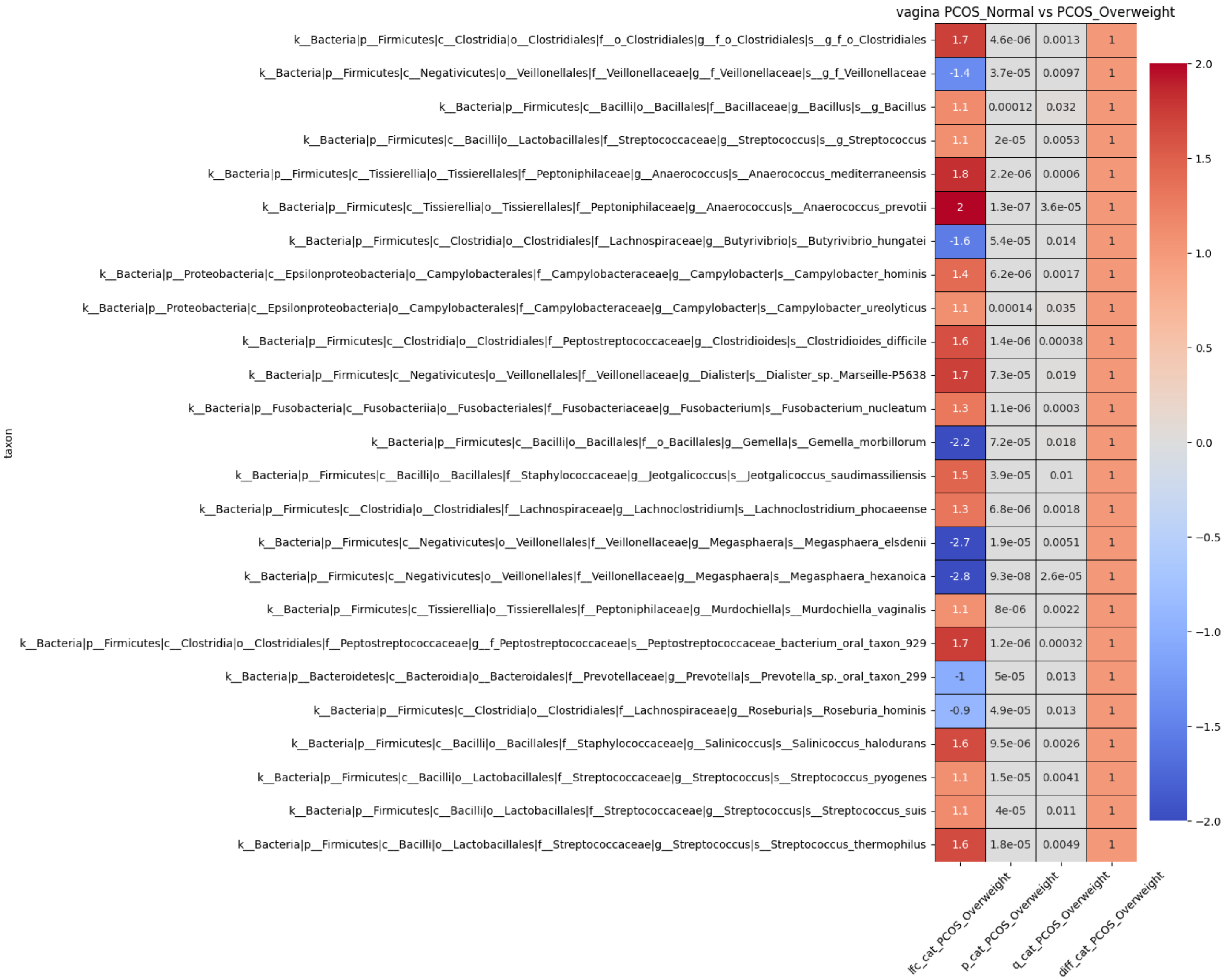

Supplementary Figure 6. ANCOM-BC differential abundance analysis of vaginal taxa between normal-weight women with PCOS and overweight women with PCOS. Only significantly different taxa (FDR-adjusted q<0.05) are shown

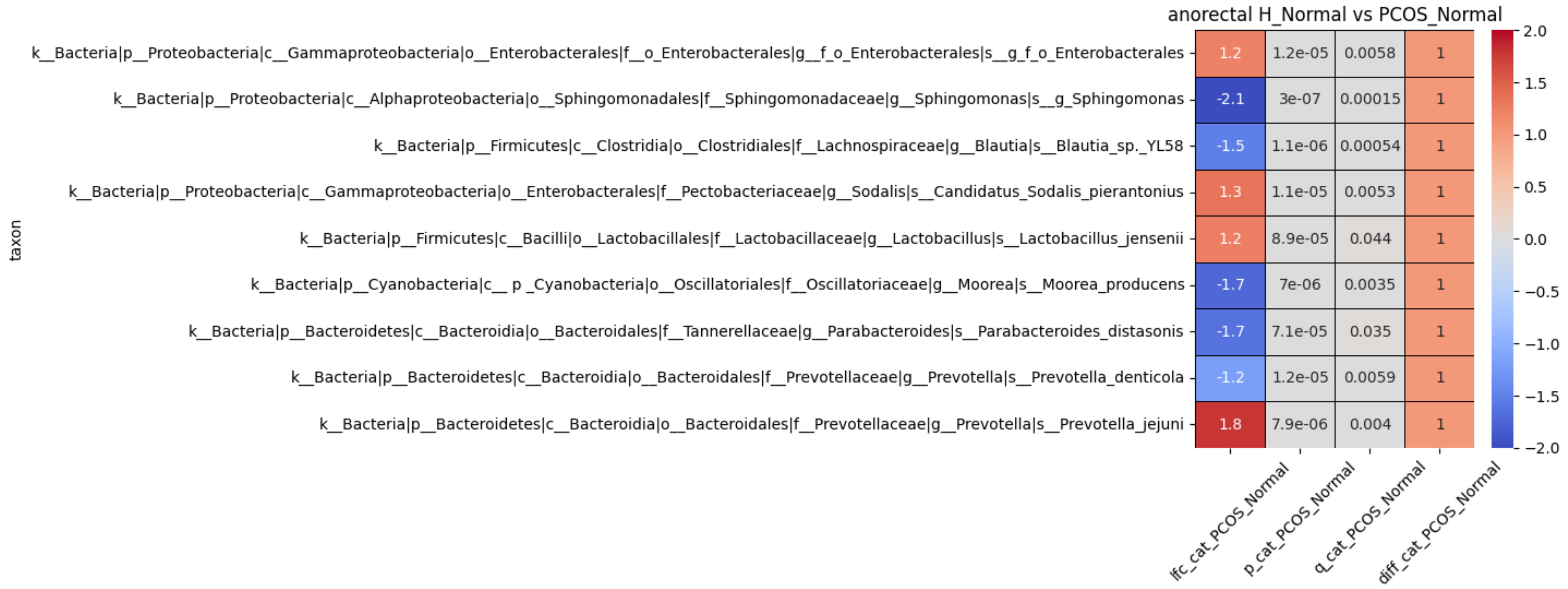

Supplementary Figure 7. ANCOM-BC differential abundance analysis of gut taxa between normal-weight healthy women and normal-weight women with PCOS. Only significantly different taxa (FDR-adjusted q<0.05) are shown.

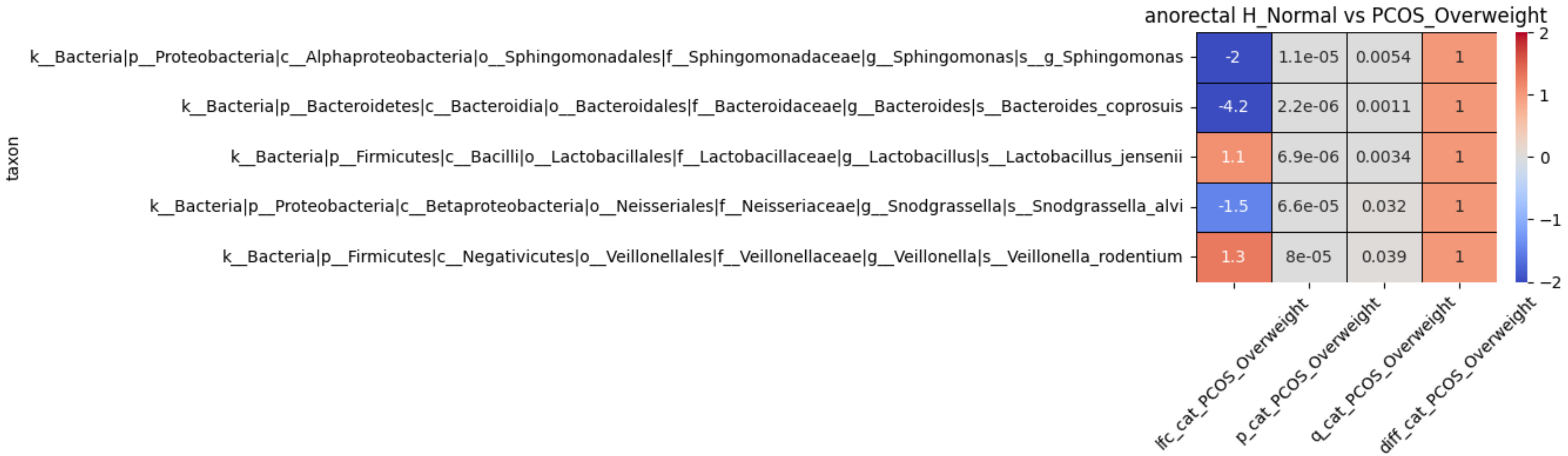

Supplementary Figure 8. ANCOM-BC differential abundance analysis of gut taxa between normal-weight healthy women and overweight women with PCOS. Only significantly different taxa (FDR-adjusted q<0.05) are shown.

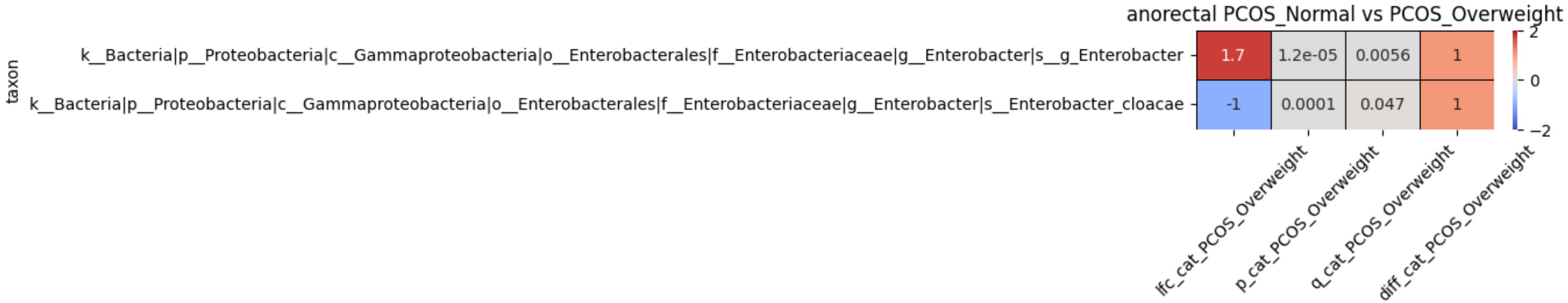

Supplementary Figure 9. ANCOM-BC differential abundance analysis of gut taxa between normal-weight women with PCOS and overweight women with PCOS. Only significantly different taxa (FDR-adjusted q<0.05) are shown.
